# Symptoms of depression and anxiety, and unmet healthcare needs in adults during the COVID-19 pandemic: a cross-sectional study from the Canadian Longitudinal Study on Aging

**DOI:** 10.1186/s12889-022-14633-4

**Published:** 2022-12-01

**Authors:** Jayati Khattar, Lauren E. Griffith, Aaron Jones, Vanessa De Rubeis, Margaret de Groh, Ying Jiang, Nicole E. Basta, Susan Kirkland, Christina Wolfson, Parminder Raina, Laura N. Anderson, the Canadian Longitudinal Study on Aging (CLSA) Team, Andrew Costa, Cynthia Balion, Yukiko Asada, Benoȋt Cossette, Melanie Levasseur, Scott Hofer, Theone Paterson, David Hogan, Jacqueline McMillan, Teresa Liu-Ambrose, Verena Menec, Philip St. John, Gerald Mugford, Zhiwei Gao, Vanessa Taler, Patrick Davidson, Andrew Wister, Theodore Cosco

**Affiliations:** 1 Department of Health Research Methods, Evidence, and Impact, McMaster University, 1280 Main St. West, Hamilton, Ontario L8S 4L8 Canada; 2 Applied Research Division, Center for Surveillance and Applied Research, Public Health Agency of Canada, Ottawa, ON K0A 0K9 Canada; 3 Department of Epidemiology, Biostatistics and Occupational Health, School of Population and Global Health, McGill University, Montreal, Canada; 4 Department of Community Health & Epidemiology and Division of Geriatric Medicine, Dalhousie University, Halifax, Canada; 5 Department of Epidemiology, Biostatistics and Occupational Health, School of Population and Global Health & Department of Medicine, McGill University, Montreal Canada & Research Institute of the McGill University Health Centre, McGill University, Montreal, Canada

**Keywords:** Depression, Anxiety, Unmet healthcare needs, CLSA

## Abstract

**Background:**

The COVID-19 pandemic disrupted access to healthcare services in Canada. Research prior to the pandemic has found that depression and anxiety symptoms were associated with increased unmet healthcare needs. The primary objective of this study was to examine if mental health was associated with perceived access to healthcare during the pandemic

**Methods:**

A cross-sectional study was conducted using data from 23,972 participants (aged 50-96) in the Canadian Longitudinal Study on Aging COVID-19 Exit Survey (Sept-Dec 2020). We used logistic regression to estimate how the presence of depression and anxiety symptoms, defined using scores of ≥10 on the Center for Epidemiologic Studies Depression Scale and ≥10 on the Generalized Anxiety Disorder Scale, were associated with the odds of reporting: 1) challenges accessing healthcare, 2) not going to a hospital or seeing a doctor when needed, 3) experiencing barriers to COVID-19 testing. Models were adjusted for sex, age, region, urban/rural residence, racial background, immigrant status, income, marital status, work status, chronic conditions, and pre-pandemic unmet needs.

**Results:**

The presence of depressive (aOR=1.96; 95% CI=1.82, 2.11) and anxiety symptoms (aOR=2.33; 95% CI=2.04, 2.66) compared to the absence of these symptoms were independently associated with higher odds of challenges accessing healthcare. A statistically significant interaction with sex suggested stronger associations in females with anxiety. Symptoms of depression (aOR=2.88; 95% CI=2.58, 3.21) and anxiety (aOR=3.05; 95% CI=2.58, 3.60) were also associated with increased odds of not going to a hospital or seeing a doctor when needed. Lastly, depressive (aOR=1.99; 95% CI=1.71, 2.31) and anxiety symptoms (aOR=2.01; 95% CI=1.58, 2.56) were associated with higher odds of reporting barriers to COVID-19 testing. There was no significantly significant interaction with sex for the latter two outcomes.

**Conclusion:**

The presence of depression and anxiety symptoms were strongly associated with perceived unmet healthcare needs during the COVID-19 pandemic. Interventions to improve healthcare access for adults with depression and anxiety during the pandemic may be necessary.

**Supplementary Info﻿rmation:**

The online version contains supplementary material available at 10.1186/s12889-022-14633-4.

## Background

Due to the onset of the COVID-19 pandemic in March 2020, various public health restrictions were implemented in all Canadian provinces and territories [1]. As part of the public health response, many healthcare system resources were re-directed to caring for COVID-19 patients and several services switched to virtual delivery [2]. This led to a disruption in the delivery of primary care, hospital and home-based services [3–5]. Fear of COVID-19 and cancellation or delay of appointments also led to challenges accessing services [6, 7]. From March 2020 to May 2021, 49% of Canadians reported difficulty accessing healthcare services, and 15% did not receive required healthcare services [8]. In the Canadian Longitudinal Study on Aging (CLSA), 25% of middle-aged and older adults reported challenges accessing healthcare and 8% reported that when they did not visit a hospital or doctor, while needing to, during the second wave of the COVID-19 pandemic (Sept. – Dec. 2020) [9]. Longitudinal studies have found that unmet healthcare needs can contribute to deterioration in future health and quality of life [10–12], therefore it is critical to identify populations that experienced greater levels of unmet need during the COVID-19 pandemic and implement interventions to reduce potential impacts.

Prior to the pandemic, research has found interconnections between depression, anxiety and unmet healthcare needs. Multiple studies have found that experiencing symptoms of depression is associated with elevated unmet needs and vice versa [13–16]. In a cross-sectional study of 845 older adults in Germany, the odds of experiencing depressive symptoms were 82% higher in the group that had unmet needs [17]. People with symptoms of anxiety are also more likely to report unmet needs [18–20], with one study of HIV patients finding that those with anxiety were two times more likely to miss a follow-up appointment [21]. Studies in this field have found similar results during the pandemic, with cross-sectional studies from numerous countries, such as the United States [22, 23], Netherlands [24] and Germany [25], finding that those experiencing symptoms of depression and anxiety were more likely to report unmet healthcare needs. Further, individuals experiencing mental distress during the pandemic were significantly more likely to report delaying medical appointments and prescription appointments [26, 27]. Overall, the research to date suggests that individuals with mental health conditions faced greater challenges accessing healthcare services during the pandemic.

In Canada, there were several changes to the healthcare system in response to the COVID-19 pandemic, including an increase in virtual visits and changes to the availability of in-person services. Furthermore, the Canadian population experienced a mental health crisis through the pandemic. The prevalence of major depressive disorder increased from 6.7% to 16.3% during the fall of 2020 among adults aged 18+ (Sept. – Dec. 2020) [28]. Similarly, the prevalence of generalized anxiety disorder in the population also increased from 2.5% [29] to 13% during the same time period [30]. Research from the CLSA found that 43% of middle aged and older adults in Canada experienced a worsening of depression symptoms during the pandemic [31]. Other national surveys provide evidence that symptoms of depression and anxiety worsened in Canadian adults during this time [32, 33]. It is critical to examine the unmet healthcare needs of middle and older aged adults during the first year of the pandemic and to assess the unmet healthcare needs of individuals with mental health conditions.

Concerningly, studies conducted both before and during the pandemic have found that women have reported greater levels of unmet need, relative to men [34, 35]. Women have also been more likely to report symptoms of depression and anxiety, relative to men [36, 37]. Some research has examined how sex and gender may interact with certain socioeconomic characteristics, such as marital status and education, to result in different levels of unmet healthcare needs [38, 39]. However, there has been limited attention given to potential interactions with depression and anxiety. The COVID-19 pandemic has been acknowledged to have gendered impacts, with women experiencing greater levels of unemployment [40] and caregiving responsibilities [41]. Examining the interaction of depression and anxiety with sex may give insight into the pathways through which mental health affected healthcare needs during the pandemic.

There is currently a research gap in the literature, with few studies examining the Canadian experience of unmet healthcare needs during the COVID-19 pandemic. Therefore, the primary objective of this study was to examine the association between symptoms of depression and anxiety and unmet healthcare needs among middle aged and older adults. As a secondary objective, the interaction between depression, anxiety and sex was examined to assess if the association between mental health and unmet needs is modified by sex.

## Methods

### Study design & data source

A cross sectional study was conducted using data from the CLSA COVID-19 exit survey (Sept. – Dec. 2020). The CLSA is a national cohort study that recruited 51,338 adults from across the ten provinces [42]. Recruitment and baseline data collection occurred between 2011 to 2015 and follow-up one (FUP1) occurred from 2015 to 2018 (n=44,817). Participants will be followed up every three years until at least 2033. During recruitment at baseline (2011-2015), participants were required to be between the ages of 45 to 85, able to complete interviews in French or English. Recruitment excluded residents of the three territories, First Nations Reserves or settlements, any institutions (e.g., long term care homes) and full-time members of the Armed Forces due to difficulty establishing and maintaining contact. The study also excluded individuals with signs of cognitive impairment [43]. The CLSA has been extensively described by Raina et al. and information on the study is also available online (https://www.clsa-elcv.ca/) [31, 43, 44]. Ethics approval was granted by the Hamilton Integrated Research Ethics Board and from each data collection site across Canada.

In response to the COVID-19 pandemic, the CLSA COVID-19 Questionnaire Study was launched. Participants who could be contacted were invited to participate in the study via web or telephone surveys. There were 42,511 participants invited and 28,559 (response rate=67%) completed the baseline survey, which was administered between April 15^th^, 2020 and May 30^th^, 2020. Participants then completed weekly/bi-weekly/monthly surveys until the final COVID-19 exit survey was administered between September 29^th^, 2020 and December 29^th^, 2020. While 24,114 participants completed the CLSA COVID-19 exit survey, 23,975 participants had data available from the CLSA baseline, FUP1 and COVID-19 surveys. Three participants were excluded from the sample as they lived in one of the three territories in 2020, resulting in a final sample of 23,972 adults for this analysis.

### Measurement of depression and anxiety

The main independent variables of interest were self-reported symptoms of depression and anxiety assessed at the time of the CLSA COVID-19 exit survey (Sept. – Dec. 2020) [31]. Depressive symptoms were evaluated using the 10-item version of the Center for Epidemiologic Studies Depression Scale (CESD-10). Participants are asked to report the frequency of experiencing certain feelings or behaviours within the past week on a four-point Likert scale, ranging from “All of the time” to “Rarely or never”. CESD-10 composite scores range from 0 to 30. In this study, we have categorized scores ≥10 as indicative of depression symptoms based on the scale’s guidelines [45]. The CESD-10 has shown good validity and reliability [46–48]. Symptoms of anxiety were assessed using the Generalized Anxiety Disorder 7 (GAD-7) scale. The GAD-7 consists of seven items, with participants asked to rank how frequently in the past two weeks they felt bothered by given concerns on a four-point Likert scale from “Not at all” to “Nearly every day”. GAD-7 composite scores range from 0 to 21. We used a cut off score of ≥10 to indicate the presence of anxiety symptoms, as has been suggested by the scale’s developers and supported by subsequent validation studies [49, 50]. The GAD-7 scale has also shown a high level of reliability [51, 52]. While the CESD-10 and GAD-7 have been validated for use with Canadian adolescents [53, 54], they have not been extensively tested for use with the Canadian adult population. However, both scales are well-supported in the literature for use with middle-aged and older adults [55–57]. The GAD-7 was used by Statistics Canada in their Canadian Perspective Survey Series, which sampled individuals aged 15+ from across the country during the pandemic [58].

### Measurement of unmet healthcare needs

Our primary outcome of interest, the self-reported experience of unmet healthcare needs, was assessed using three questions that were included in the CLSA COVID-19 exit survey: 1) “Since the beginning of the COVID-19 pandemic have you experienced any challenges accessing healthcare?” 2) “Since March 1^st^, 2020 were there times when you did not go to the hospital or to see a doctor even though you needed to?”, and 3) “Since the beginning of the COVID-19 pandemic, have you experienced barriers to accessing testing for COVID-19?” [9]. For each question, participants could respond by saying “Yes”, “No”, “Don’t know / No answer” or “Prefer not to answer”. It is recognized that the questions do not differentiate between individuals who did or did not perceive a need for healthcare services during the pandemic. As a consequence, individuals that indicated “No” to the questions may have either not perceived a need for healthcare services at all or were satisfied with the services they received. These questions were not formally validated prior to administration of the survey but resemble the questions asked in the Canadian Community Health Survey (CCHS) [34] and European Union Statistics on Income and Living Conditions (EU-SILC) [59].

### Confounding variables

Potential confounding variables were selected *a priori* and defined as variables that were a potential risk factor of the outcome, associated with the exposures, and not on the causal pathway between the exposure and the outcome. These included: sex, age, geographic region, urban/rural, racial background, immigrant status, household income, marital status, work status, chronic conditions and pre-pandemic unmet needs. Sex, racial background and immigrant status were assessed at CLSA baseline (2011-2015). Participants were classified as immigrants if they were not born in Canada. Household income, marital status and pre-pandemic unmet needs were measured at CLSA FUP1 (2015-2018). At FUP1, pre-pandemic unmet needs were assessed by asking participants if “During the past 12 months, was there ever a time when you felt that you needed healthcare but you didn’t receive it?”. Age, geographic region (Atlantic: Prince Edward Island, Nova Scotia, New Brunswick, Newfoundland; Quebec; Ontario; Prairies: Manitoba, Saskatchewan, Alberta; British Columbia), urban/rural residence, work status and presence of chronic conditions were measured from the COVID-19 baseline survey. Participants were classified as living in urban or rural areas, by linking their postal code to the Statistics Canada Postal Code Conversion file. Work status was classified as not working outside of their residence or working outside of their residence, whether as an essential worker or a non-essential worker. Participants were categorized as having a chronic condition if they reported that over the course of their lifetime a doctor had ever diagnosed them with of the following conditions: chronic obstructive pulmonary disease, other chronic lung diseases, diabetes, high blood pressure, heart disease, cancer, heart/lung/kidney/liver/pancreas failure, autoimmune disorder, pneumonia and human immunodeficiency virus. If they reported having any of the illnesses, they were considered to have a chronic condition. During development of the COVID-19 baseline survey, these conditions were chosen as they represented an elevated COVID-19 mortality risk.

### Statistical analyses

Descriptive statistics, including the frequency and distribution of all variables, were first calculated for the overall sample of 23,972 participants. Descriptive statistics are also provided for the sample stratified by the presence of symptoms of depression and anxiety. Odds ratios (OR) and 95% confidence intervals (CI) were estimated from logistic regression models. Models were constructed to assess the association between symptoms of depression and anxiety and each of the three unmet healthcare need outcomes, separately. Unadjusted estimates were first obtained and then fully adjusted logistic regression models were run to obtain adjusted odds ratios (aOR) and 95% CIs. The fully adjusted models included all potential confounders identified *a priori*: sex, age, geographic region, urban/rural, racial background, immigrant status, household income, marital status, work status, chronic conditions, and pre-pandemic unmet needs.

To fulfill the secondary objective, the models were tested for the interaction of depression and anxiety with sex. The p-values for the interaction coefficients are reported, with stratified analyses presented by sex. Sensitivity analyses were conducted to test the interaction of depression and anxiety with pre-pandemic unmet needs. This was done to assess if the associations between the mental health exposures and unmet need outcomes differed between those who already expressed unmet need prior to the pandemic, compared to those who did not. As a part of the sensitivity analysis, we also calculated the aORs for the regression models that adjusted for all covariates except for pre-pandemic unmet needs. A sensitivity analysis was also done to test the interaction of depression and anxiety with age. To evaluate if the co-occurrence of depression and anxiety affected the results, depression and anxiety were also included simultaneously in the models. The co-occurrence of the outcomes of challenges accessing healthcare and not going to a hospital or seeing a doctor when needed was also evaluated.

The software SAS v9.4 was used to perform the statistical calculations. There was relatively minimal missing data (i.e., less than 10% for any variable), and only cases with complete data on all variables were included in the regression models and therefore no imputation was performed. The threshold of statistical significance was p-value <0.05.

## Results

The descriptive characteristics of the 23,972 adults eligible for this analysis are summarized in Table 1. The majority of the sample is aged 65 or above (65.2%). We found that 22% (N=5179) of participants screened positive for symptoms of depression and 5% (N=1176) of participants screened positive for symptoms of anxiety in the COVID-19 exit survey (Sept. – Dec. 2020). The Cronbach’s alphas for the CESD-10 and GAD-7 were 0.85 and 0.84, respectively. Regarding unmet healthcare needs, 25% (N=5992) of participants reported challenges accessing healthcare, 8% (N=1776) reported not visiting the hospital or seeing the doctor while needing to and 4% (N=917) reported barriers to accessing testing for COVID-19.

**Table 1:** Descriptive characteristics of the sample at CLSA COVID-19 exit (Sept. – Dec. 2020)

	COVID-19 Exit Survey (N=23972) N (%)	CES-D 10 Score ≥10 (N=5179) N (%)	GAD-7 Score ≥10 (N=1176) N (%)
**Sex**			
Female	12743 (53.2)	3295 (63.6)	778 (66.2)
Male	11229 (46.8)	1884 (36.4)	398 (33.8)
**Age**			
50-54	1097 (4.6)	312 (6.0)	111 (9.4)
55-64	7250 (30.2)	1664 (32.2)	486 (41.3)
65-74	8759 (36.5)	1777 (34.3)	365 (31.1)
75-84	5145 (21.5)	1042 (20.1)	169 (14.4)
85-96	1721 (7.2)	384 (7.4)	45 (3.8)
**Geographic region**			
Atlantic	4334 (18.1)	855 (16.5)	194 (16.5)
Prairies	5130 (21.4)	1151 (22.2)	268 (22.8)
Ontario	5554 (23.2)	1264 (24.4)	288 (24.5)
Quebec	4336 (18.1)	823 (15.9)	180 (15.3)
British Columbia	4618 (19.3)	1086 (21.0)	246 (20.9)
**Urban/Rural**			
Rural area	4245 (17.8)	790 (15.3)	197 (16.8)
Urban area	19602 (82.2)	4368 (84.7)	975 (83.2)
Missing	125	21	4
**Racial background**			
White	23273 (97.2)	5033 (97.3)	1133 (96.5)
Non-white	673 (2.8)	140 (2.7)	41 (3.5)
Missing	26	6	2
**Immigrant status**			
Immigrant	3789 (15.8)	822 (15.9)	176 (15.0)
Non-immigrant	20173 (84.2)	4356 (84.1)	1000 (85.0)
Missing	10	1	0
**Household income**			
Less than $20,000	861 (3.8)	264 (5.5)	66 (6.0)
$20,000 to <$50,000	4855 (21.5)	1242 (25.6)	265 (24.1)
$50,000 to <$100,000	8569 (37.9)	1803 (37.2)	384 (34.9)
$100,000 to <$150,000	4589 (20.3)	878 (18.1)	226 (20.6)
$150,000 or more	3758 (16.5)	660 (13.6)	158 (14.4)
Missing	1340	332	77
**Marital status**			
Single, never married or never lived with a partner	2007 (8.4)	562 (10.9)	112 (9.5)
Married or living with a partner	16833 (70.3)	3256 (62.9)	808 (68.8)
Widowed	2332 (9.7)	555 (10.7)	88 (7.5)
Divorced or separated	2785 (11.6)	802 (15.5)	167 (14.2)
Missing	15	4	1
**Chronic conditions**			
Present	14235 (59.7)	3304 (64.3)	740 (63.3)
Absent	9594 (40.3)	1837 (35.7)	429 (36.7)
Missing	143	38	7
**Work status**			
Does not work outside the house	17357 (74.6)	3679 (73.7)	743 (66.1)
Essential worker	2495 (10.7)	510 (10.2)	149 (13.3)
Non-essential worker	3426 (14.7)	801 (16.1)	232 (20.6)
Missing	694	189	52
**Unmet needs (pre-pandemic)**			
Yes	1874 (7.8)	685 (13.3)	199 (16.9)
No	22060 (92.2)	4485 (86.7)	976 (83.1)
Missing	38	9	1
**Challenges in accessing healthcare (pandemic)**			
Yes	5992 (25.3)	1898 (36.8)	519 (44.3)
No	17759 (74.7)	3261 (63.2)	652 (55.7)
Missing	221	20	5
**Not going to a hospital or seeing a doctor when needed (pandemic)**			
Yes	1776 (7.5)	763 (14.8)	239 (20.4)
No	21989 (92.5)	4401 (85.2)	933 (79.6)
Missing	207	15	4
**Experienced barriers to accessing testing for COVID-19 (pandemic)**			
Yes	917 (3.9)	331 (6.4)	94 (8.0)
No	22828 (96.1)	4830 (93.6)	1077 (92.0)
Missing	227	18	5

Descriptive characteristics of the sample with symptoms of depression and anxiety can be found in Table 1. Females represented a greater proportion of those with depression and anxiety symptoms. Individuals with chronic conditions and pre-pandemic unmet needs also represented a substantially greater proportion of those with symptoms, relative to those without. Participants that reported working outside of the home, whether as an essential or non-essential worker, comprised a greater proportion of those that had anxiety symptoms, compared to those that did not work outside of the home.

As shown in Table 2, the presence of both depressive and anxiety symptoms were strongly associated with all three unmet healthcare outcomes. The fully adjusted results were similar to the unadjusted results, with the aORs only slightly attenuated. Depressive symptoms (aOR=1.96; 95% CI=1.82, 2.11) and anxiety (aOR=2.33; 95% CI=2.04, 2.66) were strongly associated with increased odds of reporting challenges accessing the healthcare system. Similarly, symptoms of depression (aOR=2.88; 95% CI=2.58, 3.21) and anxiety (aOR=3.05; 95% CI=2.58, 3.60) were associated with increased odds of not going to a hospital or seeing a doctor when needed. Lastly, both depression (aOR=1.99; 95% CI=1.71, 2.31) and anxiety (aOR=2.01; 95% CI=1.58, 2.56) were associated with increased odds of reporting experiencing barriers to accessing testing for COVID-19.

**Table 2:** Logistic regression models examining depression and anxiety with unmet ﻿healthcare needs (Sept.–Dec. 2020)

Challenges in accessing healthcare	Unadjusted Model		Adjusted Model	
	OR (95% CI)	Sample Size (N)	aOR (95% CI)^a^	Sample Size (N)
**Depression**				
Negative	Reference	23,570	Reference	21,476
Positive	2.07 (1.93, 2.21)		1.96 (1.82, 2.11)	
**Anxiety**				
Negative	Reference	23,059	Reference	21,024
Positive	2.49 (2.21, 2.81)		2.33 (2.04, 2.66)	
**Not going to a hospital or seeing a doctor when needed**				
**Depression**				
Negative	Reference	23,583	Reference	21,488
Positive	3.03 (2.74, 3.35)		2.88 (2.58, 3.21)	
**Anxiety**				
Negative	Reference	23,072	Reference	21,037
Positive	3.54 (3.04, 4.12)		3.05 (2.58, 3.60)	
**Experiencing barriers to accessing testing for COVID-19**				
**Depression**				
Negative	Reference	23,565	Reference	21,473
Positive	2.10 (1.83, 2.41)		1.99 (1.71, 2.31)	
**Anxiety**				
Negative	Reference	23,057	Reference	21,023
Positive	2.32 (1.86, 2.90)		2.01 (1.58, 2.56)	

^a^Adjusted for sex, age, geographic region, urban/rural, racial background, immigrant status, income, marital status, work status, chronic conditions, unmet needs (pre-pandemic)

### Sex stratification

A statistically significant interaction was observed between sex and anxiety (p=0.016) for the outcome of reported challenges accessing the healthcare system. The results were significant for both males and females, but the association of reported challenges accessing healthcare was slightly elevated in females with symptoms of anxiety. The results were elevated and significant for depression and anxiety in both males and females for the other two outcomes, but no statistically significant interactions with sex were observed. The sex-stratified results for all of the outcomes are shown in Table 3.

**Table 3:** Adjusted logistic regression models for experiencing unmet healthcare needs, stratified by sex (Sept. – Dec. 2020)

	Males		Females		Interaction term
	aOR (95% CI)^a^	Sample Size (N)	aOR (95% CI)^a^	Sample Size (N)	*P*-value^b^
**Challenges in accessing healthcare**					
**Depression**					
Negative	Reference	10,244	Reference	11,232	0.075
Positive	1.84 (1.64, 2.06)		2.05 (1.87, 2.56)		
**Anxiety**					
Negative	Reference	10,103	Reference	10,921	0.016
Positive	1.91 (1.53, 2.39)		2.59 (2.20, 3.05)		
**Not going to a hospital or seeing a doctor when needed**					
**Depression**					
Negative	Reference	10,247	Reference	11,241	0.718
Positive	2.82 (2.37, 3.35)		2.92 (2.53, 3.37)		
**Anxiety**					
Negative	Reference	10,107	Reference	10,930	0.568
Positive	3.22 (2.42, 4.27)		2.99 (2.43, 3.68)		
**Experiencing barriers to accessingtesting for COVID-19**					
**Depression**					
Negative	Reference	10,235	Reference	11,238	0.124
Positive	1.74 (1.39, 2.19)		2.21 (1.80, 2.71)		
**Anxiety**					
Negative	Reference	10,096	Reference	10,927	0.272
Positive	1.70 (1.13, 2.59)		2.19 (1.62, 2.96)		

^a^Adjusted for age, geographic region, urban/rural, racial background, immigrant status, income, marital status, work status, chronic conditions, unmet needs (pre-pandemic)

^b^While aORS were calculated using stratified samples, the interaction term was calculated using the full, unstratified sample sizes shown in Table 2 for the adjusted models

### Sensitivity analyses

Testing for interaction between pre-pandemic unmet needs and the mental health exposures did not result in any statistically significant outcomes (Additional file 1 Supplementary Table 1). Therefore, the association between depression, anxiety and unmet healthcare needs during the pandemic did not differ between those with and without pre-pandemic unmet needs. The aORs for the models excluding pre-pandemic unmet needs are similar to the aORs for the models that include them (Additional file 1 Supplementary Table 2). Testing for interaction of age with depression and anxiety did not result in statistically significant interaction values (Additional file 1 Supplementary Table 3). Notably, of those with anxiety symptoms, 90.2% of the participants also had depressive symptoms (Additional file 1 Supplementary Table 4). When including both depression and anxiety in the fully adjusted logistic regression models, the aORs for anxiety were attenuated but the aORs for depression changed minimally (Additional file 1 Supplementary Table 5). Notably, 19.8% of those who reported challenges accessing healthcare also reported that they did not go to a hospital or see a doctor when needed (Additional file 1 Supplementary Table 6).

## Discussion

The results of our study suggest that the presence of depression and anxiety symptoms were strongly associated with increased unmet health care needs among adults in Canada early in the second wave of the COVID-19 pandemic. A statistically significant interaction was found for sex with anxiety for the outcome of experiencing challenges accessing healthcare, such that the association was stronger in females. Nonetheless, the association between mental health challenges and challenges accessing healthcare services was also strong in males. No significant interaction by sex was observed for the other two outcomes.

Our results are consistent with other studies conducted prior to and during the pandemic, which have repeatedly found that depression and anxiety symptoms are associated with elevated levels of unmet healthcare needs [23, 24, 60, 61]. Numerous factors have been proposed to explain why individuals with symptoms of these mental health illnesses consistently report higher levels of unmet need. Symptoms of the conditions themselves may make it difficult for individuals to seek services. Common symptoms of depression include self-neglect and lack of self-efficacy, which can decrease capacity to seek care [62, 63]. Individuals with anxiety may avoid seeking healthcare due to fear of being diagnosed with an illness or discomfort with unfamiliar environments [18]. Additionally, individuals with these mental health conditions may experience difficulties navigating the healthcare system. Previous studies have found that people with depression and anxiety are more likely to report barriers to care [64] and lower levels of satisfaction with care [65, 66]. Furthermore, depression and anxiety can co-occur with other risk factors for unmet needs. Depression and anxiety have been associated with increased risk of developing physical chronic conditions [67, 68], which are a significant predictor of unmet healthcare needs [34]. Depression and anxiety are also associated with financial instability and unemployment [69], which can limit ability to seek healthcare services. Studies both prior to and during the pandemic have noted that individuals with depression and anxiety can have higher levels of healthcare utilization [18, 60, 61, 70]. Greater use of services means that individuals may have had more opportunities to interact with the healthcare system and be dissatisfied with their care.

This study is cross-sectional and we cannot determine if the COVID-19 pandemic strengthened the association of depression and anxiety with unmet healthcare needs. However, the literature suggests several possible mechanisms for how pandemic-specific factors may have exacerbated the association of depression and anxiety with unmet healthcare needs. Individuals with depression and anxiety were more likely to report social isolation [71] and they may have had limited interaction with potential caregivers or support systems. Individuals with anxiety symptoms also reported greater fear of being infected with COVID-19 [72] and lower confidence in the adaptability of healthcare systems [73]. Financial insecurity caused by unemployment during the pandemic may have further contributed to unmet healthcare needs [74]. Consumption of news related to COVID-19, which was also associated with symptoms of anxiety and depression [75], may have demotivated individuals from seeking care. Symptoms of depression and anxiety also were noticed to worsen during the pandemic, meaning that the greater severity of the conditions may have heightened challenges accessing services [31, 32, 76]. It is possible that lack of healthcare access, i.e., unmet healthcare needs, contributed to the severity of depression and anxiety symptoms during the pandemic, fueling a cycle of unmet needs. These factors may have resulted in individuals with depressive or anxiety symptoms being more likely to avoid seeking healthcare services than prior to the pandemic.

This study found a significant interaction by sex for only one of the outcomes, challenges accessing healthcare, where the association of anxiety symptoms with the outcome was stronger for females, relative to males. However, as noted earlier, differences in unmet healthcare needs by sex and gender have been found in the literature, with women typically reporting higher levels of unmet needs than men [35, 77]. Prior to the pandemic, it has been suggested that women report higher levels of unmet needs due to greater caregiving responsibilities and lower financial freedom [78, 79]. During the pandemic, the deepening of gender inequalities in caregiving and employment [80] may have challenged women with symptoms of depression and anxiety to a greater extent than men. In a Statistics Canada survey, women with symptoms of anxiety were more likely to report pandemic-related job precarity and social isolation, relative to women without symptoms [81]. In comparison, men with symptoms of anxiety did not have a significant association with either factor.

Our study was able to contribute to the literature examining the consequences of the COVID-19 pandemic. Overall, our findings support the need for interventions to help individuals with symptoms of the conditions overcome access hesitancy and service barriers. Primary care outreach-related interventions, in which providers initiate contact, have been highlighted as beneficial for vulnerable groups that may find it difficult to seek healthcare services [82]. Mental health programs that use telemedicine may also help improve continuity of care for individuals with the conditions [83, 84]. Group exercise programs have also been recommended for improving mental health, while also helping form social bonds [85].

Strengths of this study include the large sample size with a wide variety of information, validated measures of depression and anxiety symptoms, as well as measurement of unmet healthcare needs during a critical time [46, 47, 49, 52]. However, there are some potential limitations. The sample includes a very low proportion of racialized Canadians, which limits the representativeness of the results. Further research is needed to give insight into the mechanisms of how depressive and anxiety symptoms affect healthcare seeking and to evaluate changes over time, which in turn can better inform better public health strategies or interventions. Future work may adopt a longitudinal approach, examining how symptoms of depression and anxiety affect the experience of unmet healthcare needs over a significant period of time. Research is still needed to examine the consequences for unmet healthcare needs, particularly for vulnerable groups.

## Conclusion

This study found that symptoms of depression and anxiety were strongly associated with unmet healthcare needs during the second wave of the COVID-19 pandemic (Sept. – Dec. 2020) for middle aged and older adults residing in Canada. Evidence of interaction of mental health with sex was only found for one of the outcomes, challenges accessing healthcare. While this was a cross-sectional study, we discuss how several pandemic-specific factors may have contributed to the associations identified. Further attention is needed to understand how to best serve the healthcare needs of individuals with symptoms of depression and anxiety.

## Supplementary Information

**Additional file 1.** Supplementary materials

## Data Availability

Data are available from the Canadian Longitudinal Study on Aging (www.clsa-elcv.ca) for researchers who meet the criteria for access to de-identified CLSA data.
