# Supplementary material for: Symptoms of depression and anxiety, and unmet healthcare needs in adults during the COVID-19 pandemic: a cross-sectional study from the Canadian Longitudinal Study on Aging
Source: BMC Public Health. 2022 Dec 1;22:2242. doi: 10.1186/s12889-022-14633-4 (PMC9713148; doi:10.1186/s12889-022-14633-4)
Supplement: Supplementary file 1 — Additional file 1. Supplementary materials [file 12889_2022_14633_MOESM1_ESM.docx]

**Supplementary Material**

Supplementary Table 1: Adjusted logistic regression models for unmet healthcare needs, stratified by pre-pandemic unmet needs (Sept.-Dec. 2020)

|  | Absence of Pre-Pandemic Unmet Needs | | Presence of Pre-Pandemic Unmet Needs | | Interaction term |
| --- | --- | --- | --- | --- | --- |
|  | aOR (95% CI)^a^ | Sample Size (N) | aOR (95% CI)^a^ | Sample Size (N) | P-value^b^ |
| Challenges in accessing healthcare | | | | | |
| Depression  Negative  Positive | Reference  1.96 (1.82, 2.12) | 19825 | Reference  2.04 (1.65, 2.53) | 1663 | 0.898 |
| Anxiety  Negative  Positive | Reference  2.30 (1.99, 2.65) | 19336 | Reference  2.57 (1.82, 3.62) | 1613 | 0.524 |
| Not going to a hospital or seeing a doctor when needed | | | | | |
| Depression  Negative  Positive | Reference  2.95 (2.62, | 19834 | Reference  2.63 (1.99, 3.48) | 1666 | 0.412 |
| Anxiety  Negative  Positive | Reference  3.15 (2.61, 3.80) | 19346 | Reference  2.73 (1.88, 3.96) | 1616 | 0.637 |
| Experiencing barriers to accessing testing for COVID-19 | | | | | |
| Depression  Negative  Positive | Reference  2.02 (1.72, 2.37) | 19819 | Reference  1.83 (1.20, 2.78) | 1666 | 0.544 |
| Anxiety  Negative  Positive | Reference  2.12 (1.62, 2.77) | 19332 | Reference  1.67 (0.94, 2.97) | 1616 | 0.326 |

^a^ Adjusted for sex, age, geographic region, urban/rural, racial background, immigrant status, income, marital status, work status, chronic conditions

^b^ While aORS were calculated using stratified samples, the interaction term was calculated using the full, unstratified sample sizes shown in Table 2 for the adjusted models

Supplementary Table 2: Comparison of logistic regression models that include or exclude the experience of pre-pandemic unmet needs

|  | Adjusted for All Covariates | | Adjusted for All Covariates, except pre-pandemic unmet needs | |
| --- | --- | --- | --- | --- |
|  | aOR (95% CI)^a^ | Sample Size (N) | aOR (95% CI)^b^ | Sample Size (N) |
| Challenges in accessing healthcare | | | | |
| Depression  Negative  Positive | Reference  1.96 (1.82, 2.11) | 21,476 | Reference  2.04 (1.90, 2.20) | 21,482 |
| Anxiety  Negative  Positive | Reference  2.33 (2.04, 2.66) | 21,024 | Reference  2.30 (1.99, 2.65) | 21,030 |
| Not going to a hospital or seeing a doctor when needed | | | | |
| Depression  Negative  Positive | Reference  2.88 (2.58, 3.21) | 21,488 | Reference  3.03 (2.72, 3.38) | 21,494 |
| Anxiety  Negative  Positive | Reference  3.05 (2.58, 3.60) | 21,037 | Reference  3.14 (2.60, 3.79) | 21,043 |
| Experiencing barriers to accessing testing for COVID-19 | | | | |
| Depression  Negative  Positive | Reference  1.99 (1.71, 2.31) | 21,473 | Reference  2.05 (1.77, 2.38) | 21,479 |
| Anxiety  Negative  Positive | Reference  2.01 (1.58, 2.56) | 21,023 | Reference  2.12 (1.62, 2.77) | 21,029 |

^a^ Adjusted for sex, age, geographic region, urban/rural, racial background, immigrant status, income, marital status, work status, chronic conditions and unmet needs (pre-pandemic)

^b^ Adjusted for sex, age, geographic region, urban/rural, racial background, immigrant status, income, marital status, work status, chronic conditions

Supplementary Table 3: Adjusted logistic regression models for unmet healthcare needs, stratified by age group

|  | Aged 50 – 54 | | Aged 55 – 64 | | Aged 65 – 74 | | Aged 75 – 84 | | Aged 85 – 96 | | Interaction |
| --- | --- | --- | --- | --- | --- | --- | --- | --- | --- | --- | --- |
|  | aOR (95% CI)^a^ | Sample Size (N) | aOR (95% CI)^a^ | Sample Size (N) | aOR (95% CI)^a^ | Sample Size (N) | aOR (95% CI)^a^ | Sample Size (N) | aOR (95% CI)^a^ | Sample Size (N) | P-value^b^ |
| Challenges in accessing healthcare | | | | | | | | | | | |
| Depression  Negative  Positive | Reference  1.93 (1.41, 2.64) | 979 | Reference  1.94 (1.71, 2.21) | 6487 | Reference  1.96 (1.74, 2.22) | 8014 | Reference  1.99 (1.68, 2.34) | 4530 | Reference  2.10 (1.55, 2.86) | 1466 | 0.996 |
| Anxiety  Negative  Positive | Reference  2.56 (1.65, 3.96) | 962 | Reference  2.13 (1.73, 2.63) | 6377 | Reference  2.30 (1.82, 2.90) | 7841 | Reference  2.64 (1.88, 3.72) | 4408 | Reference  3.35 (1.58, 7.12) | 1436 | 0.789 |
| Not going to a hospital or seeing a doctor when needed | | | | | | | | | | | |
| Depression  Negative  Positive | Reference  2.12 (1.34, 3.35) | 979 | Reference  2.60 (2.15, 3.16) | 6497 | Reference  2.96 (2.47, 3.54) | 8013 | Reference  3.50 (2.71, 4.52) | 4533 | Reference  3.63 (2.30, 5.75) | 1466 | 0.185 |
| Anxiety  Negative  Positive | Reference  2.44 (1.37, 4.37) | 962 | Reference  2.85 (2.18, 3.74) | 6389 | Reference  2.99 (2.22, 4.01) | 7839 | Reference  4.21 (2.76, 6.42) | 4411 | Reference  5.01 (2.12, 11.85) | 1436 | 0.254 |
| Experiencing barriers to accessing testing for COVID-19 | | | | | | | | | | | |
| Depression  Negative  Positive | Reference  1.79 (1.01. 3.16) | 980 | Reference  1.98 (1.54, 2.55) | 6491 | Reference  2.02 (1.56, 2.60) | 8009 | Reference  2.38 (1.64, 3.45) | 4529 | Reference  1.39 (0.65, 2.96) | 1464 | 0.515 |
| Anxiety  Negative  Positive | Reference  1.71 (0.79, 3.71) | 963 | Reference  1.93 (1.33, 2.79) | 6381 | Reference  2.12 (1.37, 3.28) | 7836 | Reference  2.66 (1.33, 5.32) | 4408 | Reference  1.84 (0.39, 8.69) | 1435 | 0.854 |

^a^ Adjusted for sex, geographic region, urban/rural, racial background, immigrant status, income, marital status, work status, chronic conditions and unmet needs (pre-pandemic)

^b^ While aORS were calculated using stratified samples, the interaction term was calculated using the full, unstratified sample sizes shown in Table 2 for the adjusted models

Supplementary Table 4: Frequency of anxiety symptoms reported, with co-occurrence of depression symptoms (Sept.-Dec. 2020) (N=22992)

|  | **GAD-7 Score ≥10**  **(N=1172)**  N (%) | **GAD-7 Score <10**  **(N=21820)**  N (%) |
| --- | --- | --- |
| **CESD-10 Score ≥10** | 1058 (90.3) | 114 (17.9) |
| **CESD-10 Score <10** | 3903 (9.7) | 17917 (82.1) |

Supplementary Table 5: Adjusted logistic regression models for unmet healthcare needs, including depression and anxiety Sept.-Dec. 2020)

|  | Adjusted for All Covariates | | Adjusted for All Covariates and Symptoms of Depression and Anxiety | |
| --- | --- | --- | --- | --- |
|  | aOR (95% CI)^a^ | Sample Size (N) | aOR (95% CI)^b^ | Sample Size (N) |
| Challenges in accessing healthcare | | | | |
| Depression  Negative  Positive | Reference  1.96 (1.82, 2.11) | 21,476 | Reference  1.80 (1.66, 1.95) | 20,937 |
| Anxiety  Negative  Positive | Reference  2.33 (2.04, 2.66) | 21,024 | Reference  1.56 (1.36, 1.80) | 20,937 |
| Not going to a hospital or seeing a doctor when needed | | | | |
| Depression  Negative  Positive | Reference  2.88 (2.58, 3.21) | 21,488 | Reference  2.58 (2.29, 2.92) | 20,950 |
| Anxiety  Negative  Positive | Reference  3.05 (2.58, 3.60) | 21,037 | Reference  1.66 (1.38, 1.99) | 20,950 |
| Experiencing barriers to accessing testing for COVID-19 | | | | |
| Depression  Negative  Positive | Reference  1.99 (1.71, 2.31) | 21,473 | Reference  1.89 (1.60, 2.24) | 20,122 |
| Anxiety  Negative  Positive | Reference  2.01 (1.58, 2.56) | 21,023 | Reference  1.32 (1.02, 1.73) | 20,122 |

^a^ Adjusted for sex, age, geographic region, urban/rural, racial background, immigrant status, income, marital status, work status, chronic conditions, unmet needs (pre-pandemic)

^b^ Adjusted for sex, age, geographic region, urban/rural, racial background, immigrant status, income, marital status, work status, chronic conditions, unmet needs (pre-pandemic), depression and anxiety

Supplementary Table 6: Cross-tabulation of challenges accessing healthcare with not going to a hospital or seeing a doctor when needed (Sept.-Dec. 2020) (N=23738)

|  | **Reported challenges accessing healthcare**  **(N=5988)**  N (%) | **Did not report challenges accessing healthcare (N=17750)**  N (%) |
| --- | --- | --- |
| **Reported not going to a hospital or seeing a doctor when needed** | 1186 (19.8) | 587 (3.3) |
| **Did not report not going to a hospital or seeing a doctor when needed** | 4802 (80.2) | 17163 (96.7) |
